# Retraction: Pollution halo impact in context of productive capacities, energy poverty, urbanization, and institutional quality

**DOI:** 10.1371/journal.pone.0311380

**Published:** 2024-11-11

**Authors:** The PLOS ONE Editors

The reliability of this article [1] is in question because there are issues linking it to a series of submissions for which PLOS identified concerns about authorship, data integrity, and peer review.

In addition, our post-publication assessment indicated that the article [1] does not meet *PLOS ONE*’s publication criteria.

In light of these issues, the *PLOS ONE* Editors retract the article. We regret that the issues were not identified before the article was published.

All authors did not agree with the retraction.
